# Chloral Hydrate in Scarlet Fever

**Published:** 1907-04

**Authors:** 


### CHLORAL HYDRATE IN SCARLET FEVER.

In 1896 Dr. James C. Wilson, of Philadelphia, advocated routine doses of chloral hydrate in the treatment of scarlet fever, maintaining that increased diuresis, improvement of the nervous symptoms, relief of the itching, and increased comfort for the patient followed its use. Royer (Therapeutic Gazette, January 15th) reports the results of the treatment in 800 cases in the Municipal Hospital in Philadelphia, and contrasts these results with those obtained in the same hospital in 756 cases treated with the usual remedies. The drug was administered in sufficient doses to produce light somnolence, and these doses were continued for five or six days after the subsidence of the fever. Three patients died in the series of 800 treated with chloral (0.37 per cent.), and four died in the series of 756 cases treated with the usual remedies (0.52 per cent.). During the febrile period no material difference was found in the number of patients who presented nephritis in the two groups of cases. In the post-febrile period, on the other hand, 4.25 per cent. of the patients treated with routine doses of chloral had albuminuria, 3.50 per cent. had transient nephritis, and two per cent. had severe nephritis.

Among the patients treated without chloral 5.68 per cent. had albuminuria, 5.29 per cent. had transient nephritis, and 2.38 per cent. had severe nephritis.

Royer thinks that Dr. Wilson's statements as to the beneficent influence of chloral hydrate on the symptoms presented by scarlet fever patients are, on the whole, correct. He does not find that the increase of diuresis noticed by Wilson is any more marked after the administration of chloral than after that of definite quantities of water at stated intervals. The drug does not appear to exert any depressing effect upon the circulation. It ameliorates the nervous symptoms better than any remedy so far suggested, and it does allay the annoying itching. The routine administration of the drug appears to lessen the tendency to postfebrile nephritis. It would have added to the value of Dr. Royer's paper, we think, if he had been more specific in regard to the doses. The direction to administer enough chloral "to produce light somnolence" is too indefinite. A statement of the doses for patients of different ages would have been more satisfactory.—Editorial, New York Medical Journal, March 16th, 1907.

